# Higher Incidence of Chronic Thromboembolic Pulmonary Hypertension After Acute Pulmonary Embolism in Asians Than in Europeans: A Meta-Analysis

**DOI:** 10.3389/fmed.2021.721294

**Published:** 2021-10-26

**Authors:** Wenyi Pang, Zhu Zhang, Zenghui Wang, Kaiyuan Zhen, Meng Zhang, Yunxia Zhang, Qian Gao, Shuai Zhang, Xincao Tao, Jun Wan, Wanmu Xie, Zhenguo Zhai

**Affiliations:** ^1^Department of Pulmonary and Critical Care Medicine, Center of Respiratory Medicine, China-Japan Friendship Hospital, Beijing, China; ^2^National Center for Respiratory Medicine, Beijing, China; ^3^Institute of Respiratory Medicine, Chinese Academy of Medical Sciences, Beijing, China; ^4^National Clinical Research Center for Respiratory Diseases, Beijing, China; ^5^Graduate School of Peking Union Medical College, Chinese Academy of Medical Sciences and Peking Union Medical College, Beijing, China; ^6^Department of Respiratory Medicine, Capital Medical University, Beijing, China

**Keywords:** chronic thromboembolic pulmonary hypertension, pulmonary embolism, incidence, cohort study, meta-analysis

## Abstract

**Aim:** To summarize the incidence of right heart catheter diagnosed chronic thromboembolic pulmonary hypertension (CTEPH) after acute pulmonary embolism (PE) in a meta-analysis.

**Methods:** Cohort studies reporting the incidence of CTEPH after acute PE were identified *via* search of Medline, Embase, China National Knowledge Infrastructure and WanFang databases.

**Results:** Twenty-two cohort studies with 5,834 acute PE patients were included. Pooled results showed that the overall incidence of CTEPH was 2.82% (95% CI: 2.11–3.53%). Subgroup analyses showed higher incidence of CTEPH in Asians than Europeans (5.08 vs. 1.96%, *p* = 0.01), in retrospective cohorts than prospective cohorts (4.75 vs. 2.47%, *p* = 0.02), and in studies with smaller sample size than those with larger sample size (4.57 vs. 1.71%, *p* < 0.001). Stratified analyses showed previous venous thromboembolic events and unprovoked PE were both significantly associated with increased risk of CTEPH (OR = 2.57 and 2.71, respectively; both *p* < 0.01).

**Conclusions:** The incidence of CTEPH after acute PE is ~3% and the incidence is higher in Asians than Europeans. Efforts should be made for the early diagnosis and treatment of CTEPH in PE patients, particularly for high-risk population.

## Introduction

Chronic thromboembolic pulmonary hypertension (CTEPH) is a unique form of pulmonary hypertension (PH) characterized by the fibrotic transformation of a pulmonary arterial thrombus, fixed obstruction of pulmonary arteries, and high pulmonary vascular resistance (1). Clinically, patients with advanced CTEPH often have severe symptoms of right heart failure and the clinical outcomes of these patients are very poor if untreated (1–3). Current curative treatment for patients with CTEPH is mainly pulmonary endarterectomy (PEA), which has been associated with improved survival in these patients (4, 5). However, the clinical manifestations for patients with CTEPH are largely non-specific, which are difficult to be differentiated from symptoms of pulmonary embolism (PE) (6). Therefore, the early diagnosis of CTEPH remains a challenge in current clinical practice. Accordingly, it remains undetermined whether routine screening for CTEPH in patients with PE is of rationale and the incidence of CTEPH in patients after acute PE remains varying according to the previous studies (7, 8). Although two previous meta-analyses have been published to summarize the incidence of CTEPH in acute PE patients (9, 10), only studies published before 2018 were included (11–27). Some recently published trials were not included (28–32). Moreover, it remains unknown whether patient and study characteristics such as patient ethnicity, study design, or sample size could significantly affect the incidence of CTEPH. In addition, although patients with previous venous thromboembolic events (VTE) or unprovoked PE have been suggested as high-risk patients for CTEPH (33), a quantitative analysis for the odds of these risk factors have not been performed in previous meta-analyses. Therefore, in this study, we aimed to summarize the incidence of right heart catheter diagnosed CTEPH after acute PE and explore its influencing factors in an updated meta-analysis and to explore the potential influences of patient and study characteristics on the outcome.

## Materials and Methods

The systematic review and meta-analysis was designed and performed in accordance with the MOOSE (Meta-analysis of Observational Studies in Epidemiology) (34) and Cochrane's Handbook (35) guidelines.

### Literature Searching

We systematically searched PubMed, Embase, China National Knowledge Infrastructure, and WanFang databases with the terms of (1) “chronic thromboembolic pulmonary hypertension” OR “CTEPH,” (2) “pulmonary embolism” OR “PE”; and (3) “incidence” OR “risk” OR “occurrence” OR “occur” OR “mortality” OR “prognosis” OR “predict” OR “predictor” OR “prevalence” OR “epidemiology” OR “follow” OR “followed” OR “follow-up” OR “cohort.” The search was limited to human studies published in English or Chinese. We also manually screened the reference lists of original and review articles. The final literature search was performed on October 31, 2020.

### Inclusion and Exclusion Criteria

Studies that fulfill the following criteria were included: (1) published as full-length articles in English or Chinese; (2) designed as cohort studies (prospective or retrospective, with a sample size of >20 and a minimal follow-up duration of 1 year); (3) included adult patients (≥18 years of age) after acute PE validated by computed tomographic pulmonary angiography (CTPA) or ventilation-perfusion (V/Q) lung scan; and (4) reported the incidence of CTEPH diagnosed with right heart catheterization evidenced pre-capillary pulmonary hypertension (PH) and ≥1 segmental perfusion defect on CTPA or V/Q scan. According to the 2014 ESC Guidelines, the time limit of chronic PE from symptoms to diagnosis and treatment is set as more than 3 months, and previous studies have suggested that the incidence of CTEPH is much higher in patients with chronic PE than that in patients with acute PE (36). Therefore, including studies incorporating patients with chronic PE would confound the results of the meta-analysis. Moreover, if studies with overlapping participants were encountered, reports of larger sample size were included. Abstracts, reviews, pre-clinical studies, studies including chronic PE, or studies with designs other than cohort study were excluded.

### Data Extracting and Quality Evaluation

Literature search, data extraction, and quality assessment were performed by two authors independently according to the pre-defined inclusion criteria. Discrepancies were resolved by consensus. The following data regarding the characteristics of the studies were extracted: name of first author, year of publication, country where the study was conducted, sample sizes, patient characteristics, mean age, proportion of males, number of patients with previous venous thromboembolic events (VTE), number of patients with unprovoked PE, patient selection for CTEPH screening, primary test for CTEPH screening, follow-up durations, and number of patients diagnosed as CTEPH. Patient characteristics were classified as three categories according to the inclusion criteria of the original studies (9): (1) consecutive acute PE patients for studies that applied no specific exclusion criteria; (2) acute PE survivors: defined as all consecutive patients with symptomatic PE who were alive after an initial treatment period of 6 months; and (3) acute PE survivors without major comorbidity: indicates symptomatic PE who were alive after an initial treatment period of 6 months and did not have pre-defined significant cardiopulmonary diseases, cancers or rheumatologic disorders. Patients with unprovoked PE were defined as PE patients without known risk factors, such as major surgery or immobilization, active malignancy, pregnancy or in the peripartum period, and use of oral contraceptives or hormone replacement therapy. A modified Newcastle-Ottawa Scale (NOS) (37) was used to evaluate the quality of the included studies, which predominantly focused on the aspects of selection of the study groups and the ascertainment of the outcome of interest.

### Statistical Analyses

Data of incidences and their corresponding stand errors (SEs) were calculated from 95% CIs or *p*-values, and were logarithmically transformed to stabilize variance and normalized the distribution. For studies that did not report incidence data, data regarding the incident case of CTEPH and overall enrolled patients after acute PE were extracted. The Cochrane's *Q*- and *I*^2^-test were used to evaluate the heterogeneity among the included cohort studies (38). A significant heterogeneity was considered if *I*^2^ > 50%. A random effect model was used if significant heterogeneity was detected; otherwise, a fixed effect model was applied (35). Moreover, subgroup analysis was also performed to evaluate the potential influences of study and patient characteristics on the incidence of CTEPH, such as ethnicity of the patients, study design, sample size, patient characteristics, patient selection for CTEPH screening, and the primary test for CTEPH screening. Medians of the continuous variables were selected as the cut-off for stratification. Stratified analyses were performed to evaluate the risk of CTEPH in patients with previous VTE compared those without previous VTE, and in patients with unprovoked PE compared to those with provoked PE. An odds ratio (OR) with 95% confidence interval (CI) was summarized. For meta-analysis reporting the association between previous VTE, unprovoked PE and the risk of CTEPH, publication biases were assessed by funnel plots with the Egger regression asymmetry test (39). RevMan (Version 5.1; Cochrane Collaboration, Oxford, UK) and STATA software (Version 12.0; Stata Corporation, College Station, TX) were used for the statistical analyses.

## Results

### Literature Search Results

**Figure 1:**
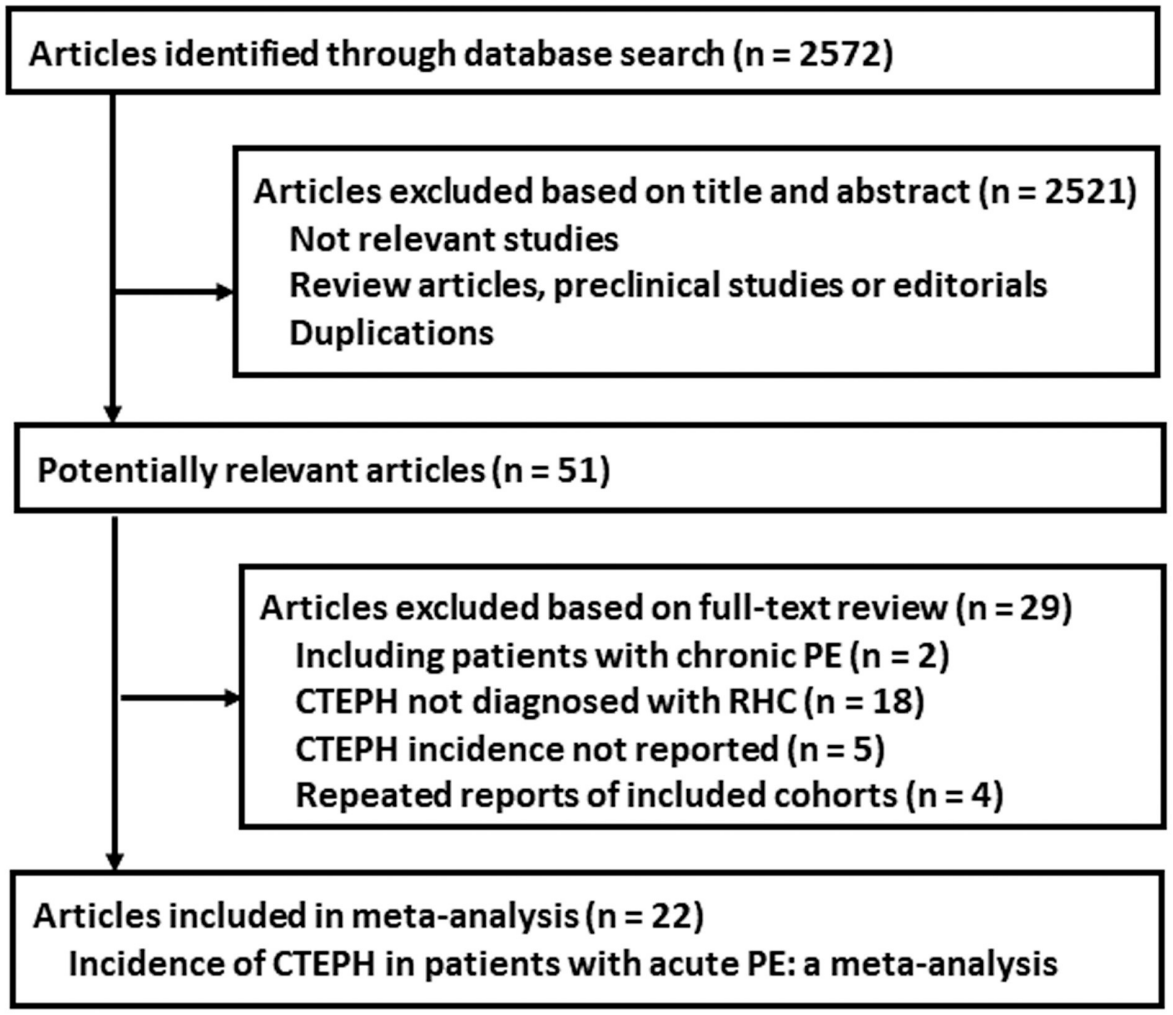
Flowchart of literature search.

The process of literature search and study identification was summarized in Figure 1. In brief, 2,572 records were identified after initial database search after excluding of the duplicated records. Further screening with titles and abstracts further excluded 2,521 records, mainly because they were irrelevant to the aim of the study. For the 51 records underwent full-text review, 29 studies were further excluded because two of them included chronic PE patients, 18 studies did not apply right heart catheter for the diagnosis of CTEPH, five did not report CTEPH incidence outcome, and the other four were repeated reports of included studies. Overall, 22 cohort studies met the inclusion criteria of the meta-analysis.

### Study Characteristics and Quality Evaluation

A total of 22 cohort studies (11–32) with 5,834 acute PE patients were included. The baseline characteristics of the included cohorts were shown in Table 1. These studies were published between 2004 and 2020, and performed in Italy (11–13, 16, 19, 28), Spain (15), the Netherlands (14, 17), Germany (18, 21, 23), France (20), Turkey (22), Czech (25), Switzerland (29), China (24, 26, 27, 31), Thailand (30), and Iran (32). Eighteen of the included studies were prospective cohort studies (11–17, 20–29, 32), while the other four were retrospective (18, 19, 30, 31). The numbers of acute PE patients included in each study varied from 43 to 866. The mean ages of the patients varied from 54 to 72 years, with the proportions of male patients ranging from 38 to 56%. With a mean follow-up 2.9 years, 174 patients were diagnosed as CTEPH. All the included patients underwent screening for CTEPH during follow-up in eight studies (14, 15, 18–20, 22–24), while the other 14 generally elected symptomatic patients for CTEPH screening (11–13, 16, 17, 21, 25–32). Most of the included studies used transthoracic echocardiography (TTE) as primary test for CTEPH screening except two studies that V/Q lung scan was applied (25, 30). Details of quality evaluation according to the modified NOS were shown in Table 2. The scales for the included studies varied between 5 and 7, demonstrating generally good study quality.

**Table 1:** Characteristics of the included cohort studies.

**References**	**Country**	**Design**	**Patient characteristics**	**Sample size**	**Mean age**	**Male**	**Patient with previous VTE**	**Patient with unprovoked PE**	**Patient selection for CTEPH**	**Primary test for CTEPH**	**Follow-up duration**	**Number of patient with CTEPH**
					**Years**	**%**					**Years**	
Pengo et al. (11)	Italy	PC	Acute PE survivors without major comorbidity	305	61.1	40.2	82	113	Symptomatic patients	TTE	7.8	18
Becattini et al. (12)	Italy	PC	Acute PE survivors without major comorbidity	259	61	44	0	135	Symptomatic patients	TTE	3.8	2
Miniati et al. (13)	Italy	PC	Consecutive acute PE patients	320	72.1	38.6	114	NR	Patients with persistent pulmonary perfusion defects	TTE	2.1	4
Martí et al. (15)	Spain	PC	Acute PE survivors without major comorbidity	110	NR	43.6	12	53	All patients	TTE	2	10
Klok (14)	The Netherlands	PC	Consecutive acute PE patients	866	55	47	195	308	All patients	TTE	2.8	4
Poli et al. (16)	Italy	PC	Acute PE survivors without major comorbidity	239	59	49.4	0	161	Symptomatic patients	TTE	3	1
Surie et al. (17)	The Netherlands	PC	Acute PE survivors without major comorbidity	110	56	45	17	NR	Symptomatic patients	TTE	3	3
Berghaus et al. (18)	Germany	RC	Acute PE survivors without major comorbidity	43	54.4	NR	43	NR	All patients	TTE	2	5
Giuliani et al. (19)	Italy	RC	Acute PE survivors without major comorbidity	111	65	41	NR	NR	All patients	TTE	1.8	5
Held et al. (21)	Germany	PC	Acute PE survivors	130	65.7	42.3	NR	NR	Symptomatic patients	TTE	2.2	8
Guérin et al. (20)	France	PC	Acute PE survivors without major comorbidity	146	61	41	35	NR	All patients	TTE	2.2	7
Kayaalp et al. (22)	Turkey	PC	Acute PE survivors without major comorbidity	99	60	44.4	1	36	All patients	TTE	1.5	5
Yang et al. (24)	China	PC	Acute PE survivors	614	61.5	48.5	0	204	All patients	TTE	3.3	10
Klok et al. (23)	Germany	PC	Acute PE survivors	134	63	56	34	76	All patients	TTE	2.5	6
Vavera et al. (25)	Czech	PC	Acute PE survivors	97	57.9	50	0	NA	Symptomatic patients	V/Q lung scan	2	4
Xi et al. (26)	China	PC	Acute PE survivors	214	61	45	0	100	Symptomatic patients	TTE	2.6	16
Xu et al. (27)	China	PC	Acute PE survivors	129	60	44.2	22	29	Symptomatic	TTE	2.2	9
Pesavento et al. (28)	Italy	PC	Acute PE survivors without major comorbidity	647	67	49.1	0	404	Symptomatic patients	TTE	3	11
Coquoz et al. (29)	Switzerland	PC	Acute PE survivors	508	61.2	53.3	144	227	Symptomatic patients	TTE	2	4
Puengpapat and Pirompanich (30)	Thailand	RC	Acute PE survivors	236	NR	NR	48	NR	Symptomatic patients	V/Q lung scan	3	12
Hsu et al. (31)	China	RC	Acute PE survivors	200	63.8	41.5	12	151	Symptomatic patients	TTE	3.8	8
Rashidi et al. (32)	Iran	PC	Acute PE survivors	317	56.5	51.3	0	21	Symptomatic patients	TTE	1	22

*VTE, thromboembolism events; PE, pulmonary embolism; CTEPH, chronic thromboembolic pulmonary hypertension; RC, retrospective cohort; PC, prospective cohort; NR, not reported; TTE, Transthoracic echocardiography; V/Q, ventilation-perfusion*.

**Table 2:** Details of study quality evaluation according to the Cochrane's Handbook guideline.

**References**	**Representativeness of the cohort**	**Confirmed diagnosis of PE**	**Reporting study protocol and all pre-specified outcomes**	**Validated assessment of outcome**	**Enough long follow-up duration**	**Adequacy of follow-up of cohorts**	**Other bias**	**Overall quality**
Pengo et al. (11)	1	1	1	1	1	1	1	7
Becattini et al. (12)	0	1	1	1	1	1	1	6
Miniati et al. (13)	1	1	0	1	0	1	1	5
Martí et al. (15)	0	1	1	1	0	1	1	5
Klok et al. (14)	1	1	1	1	0	1	1	6
Poli et al. (16)	0	1	1	1	1	1	1	6
Surie et al. (17)	0	1	1	1	1	1	1	6
Berghaus et al. (18)	0	1	1	1	0	1	1	5
Giuliani et al. (19)	0	1	1	1	0	1	1	5
Held et al. (21)	1	1	1	1	0	1	1	6
Guérin et al. (20)	0	1	1	1	0	1	1	5
Kayaalp et al. (22)	0	1	1	1	0	1	1	5
Yang et al. (24)	1	1	1	1	1	1	1	7
Klok et al. (23)	1	1	1	1	0	1	1	6
Vavera et al. (25)	1	1	1	1	0	0	1	5
Xi et al. (26)	1	1	1	1	0	1	1	6
Xu et al. (27)	1	1	1	1	0	1	1	6
Pesavento et al. (28)	0	1	1	1	1	1	1	6
Coquoz et al. (29)	1	1	1	1	0	1	1	6
Puengpapat and Pirompanich (30)	1	1	0	1	1	0	1	5
Hsu et al. (31)	1	1	1	1	1	1	1	7
Rashidi et al. (32)	1	1	1	1	0	1	1	6

### Incidence of CTEPH in Acute PE Patients

**Figure 2:**
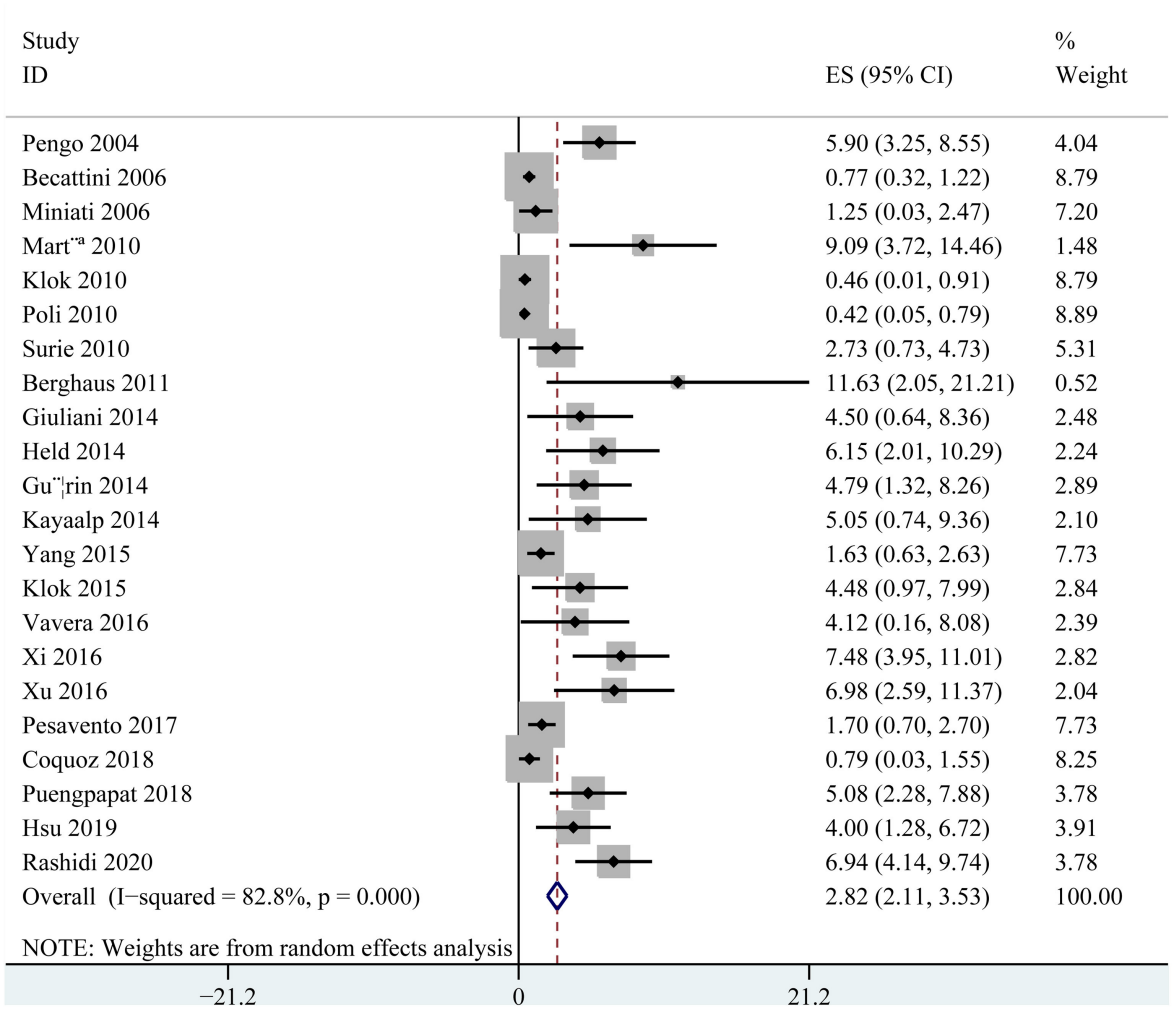
Forest plots for the meta-analysis of the incidence of CTEPH in patients after acute PE.

The incidences of CTEPH in acute PE patients varied between 0.42 and 11.63% as reported in the individual cohorts included in the meta-analysis. Significant heterogeneity was noticed (*p* for Cochrane's *Q*-test < 0.001, *I*^2^ = 82.8%). Pooling the results of the 22 cohorts showed that the overall incidence of CTEPH was 2.82% (95% confidence interval: 2.11–3.53%; Figure 2). Subgroup analyses showed higher incidence of CTEPH in Asians than Europeans (5.08 vs. 1.96%, *p* = 0.01), in retrospective cohorts than prospective cohorts (4.75 vs. 2.47%, *p* = 0.02), and in studies with smaller sample size than those with larger sample size (4.57 vs. 1.71%, *p* < 0.001; Table 3). Subgroup analyses also showed that the incidence of CTEPH was 0.64% (95% CI: 0.00–1.30%) in consecutive acute PE patients, 4.36% (95% CI: 2.72–5.99%) in acute PE survivors, and 2.60% (95% CI: 1.56–3.65%) in acute PE survivors without major comorbidity (Table 3). Screening of CTEPH in all PE patients or selected PE patients, or using TTE or V/Q lung scan as first-line test did not seem to significantly affect the reported incidence of CTEPH after acute PE (both *p* > 0.05).

**Table 3:** Subgroup analyses.

	**Incidence of CTEPH in acute PE patients**			
**Study characteristics**	**Datasets number**	**Incidence (95% CI)**	***I*^**2**^ (%)**	***P* for subgroup difference**
**Ethnicity**				
Europids	16	1.96 (1.29, 2.63)	78	
Asians	6	5.08 (2.67, 7.49)	81	0.01
**Study design**				
PC	18	2.47 (1.76, 3.18)	83	
RC	4	4.75 (3.04, 6.46)	0	0.02
**Patient characteristics**				
Consecutive acute PE patients	2	0.64 (0.00, 1.30)	29	
Acute PE survivors	10	4.36 (2.72, 5.99)	82	
Acute PE survivors without major comorbidity	10	2.60 (1.56, 3.65)	83	<0.001
**Sample size**				
>230	11	1.71 (1.04, 2.39)	84	
≤ 230	11	4.57 (3.53, 5.60)	0	<0.001
**Patient selection for CTEPH**				
All patients	8	3.56 (1.82, 5.29)	80	
Selected patients	14	2.85 (1.97, 3.74)	85	0.48
**Primary test for CTEPH**				
TTE	20	2.65 (1.93, 3.36)	83	
V/Q lung scan	2	4.76 (2.48, 7.05)	0	0.08

*PE, pulmonary embolism; CTEPH, chronic thromboembolic pulmonary hypertension; RC, retrospective cohort; PC, prospective cohort; TTE, Transthoracic echocardiography; V/Q, ventilation-perfusion; CI, confidence interval*.

### Influence of Previous VTE and Unprovoked PE on the Risk of CTEPH

**Figure 3:**
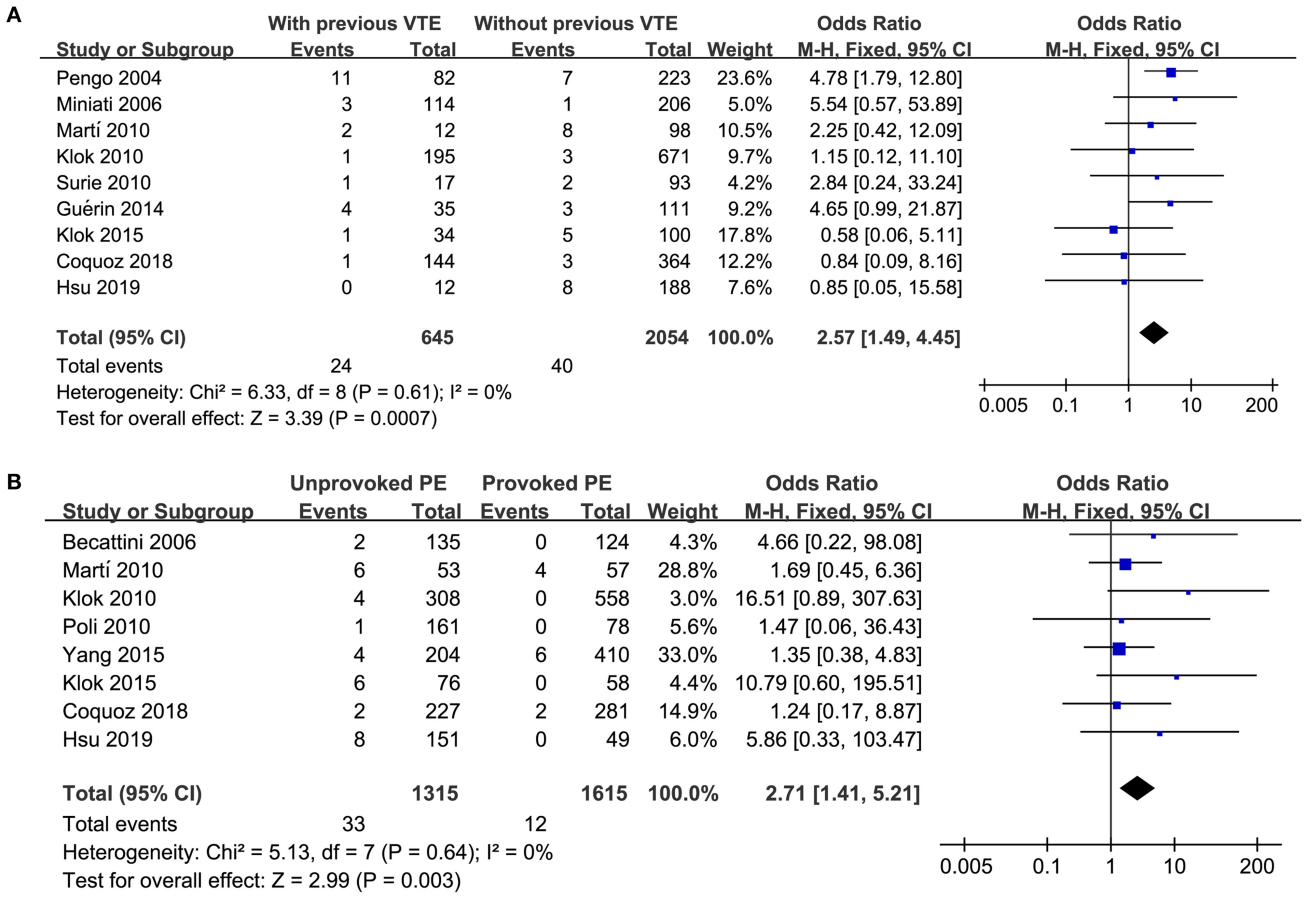
Forest plots for the meta-analysis evaluating the risk of CTEPH in patients with and without previous VTE, and in patients with provoked and unprovoked PE; **(A)** comparing risk of CTEPH in patients with and without previous VTE; and **(B)** comparing risk of CTEPH in patients with unprovoked and provoked PE.

Nine cohorts (11, 13–15, 17, 20, 23, 29, 31) reported the stratified data regarding the incidence of CTEPH in acute PE patients with or without previous VTE. Pooled results with a fixed effect model showed that previous VTE was associated with a higher risk of CTEPH in patients after acute PE (OR: 2.57, 95% CI: 1.49 and 4.45, *p* < 0.001; *I*^2^ = 0%; Figure 3A). Eight studies (12, 14, 15, 17, 23, 24, 29, 31) reported the stratified data regarding the incidence of CTEPH in provoked and unprovoked PE. Pooled results with a fixed effect model showed that patients with unprovoked PE was associated with a higher risk of CTEPH compared to those with provoked PE (OR: 2.71, 95% CI: 1.41 and 5.21, *p* = 0.003; *I*^2^ = 0%; Figure 3B).

### Publication Bias

**Figure 4:**
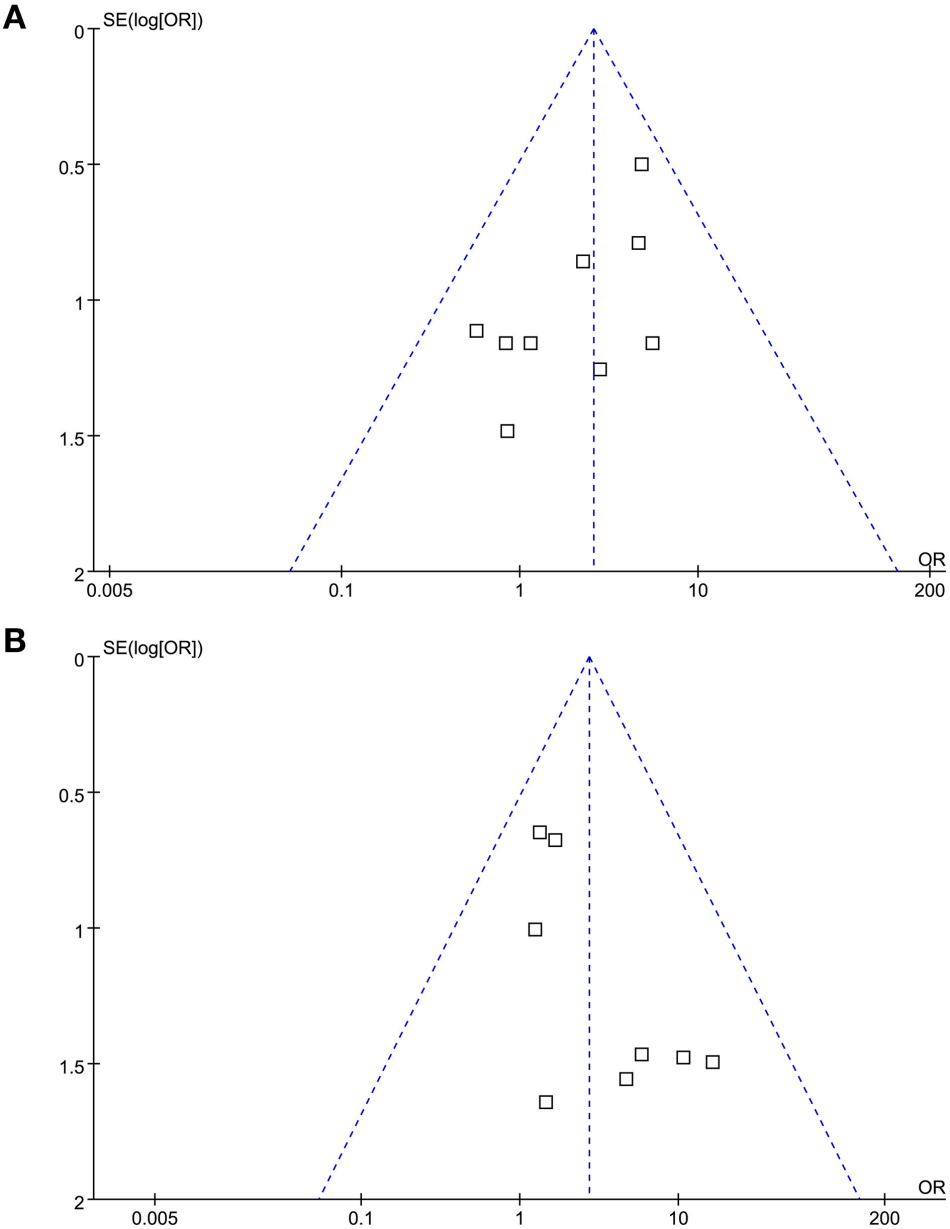
Funnel plots for the meta-analysis evaluating the risk of CTEPH in patients with and without previous VTE, and in patients with provoked and unprovoked PE; **(A)** comparing risk of CTEPH in patients with and without previous VTE; and **(B)** comparing risk of CTEPH in patients with unprovoked and provoked PE.

The funnel plot for the meta-analysis comparing the CTEPH risk in patients with and without previous VTE and in provoked and unprovoked PE were shown in Figures 4A,B. The plots were symmetrical on visual inspection, demonstrating low risk of publication bias. Results of Egger's regression-test also indicated low risk of publication bias (*p* for Egger's-test = 0.526 and 0.388, respectively).

## Discussions

In this meta-analysis of cohort studies, by pooling the results of updated cohort studies, we found an overall incidence of 2.82% for CTEPH in patients after acute PE. Subgroup analyses by patient characteristics showed that the incidence of CTEPH were 0.64% in studies with consecutive acute PE patients, 4.36% in survivors of acute PE, and 2.60% in acute PE survivors without major comorbidity. Subgroup analyses also showed higher incidence of CTEPH in Asians than Europeans (5.08 vs. 1.96%), in retrospective cohorts than prospective cohorts (4.75 vs. 2.47%), and in studies with smaller sample size than those with larger sample size (4.57 vs. 1.71%), while screening of CTEPH in all PE patients or selected PE patients, or using TTE or V/Q lung scan as first-line test did not seem to significantly affect the reported incidence. In addition, stratified analyses showed that previous venous thromboembolic events and unprovoked PE were both significantly associated with increased risk of CTEPH. Taken together, results of this updated meta-analysis showed that the incidence of CTEPH after acute PE is ~3%, and a higher incidence may exist in Asians than in Europeans, although it may be affected by patient characteristics and study design factors. Efforts should be made for the early diagnosis and treatment of CTEPH in PE patients, particularly for high-risk population.

Two previous meta-analyses have been published to summarize the incidence of CTEPH in patients after acute PE, and a comparison of cohort studies included in these two meta-analyses and those in our meta-analyses were shown in Supplementary Table 1. In 2017, the study by Ende-Verhaar et al. included 16 cohort studies published before 2016 and reported that the incidence of CTEPH diagnosed by right heart catheter was 0.56% in all comers, 3.20% in acute PE survivors, and 2.80% in acute PE survivors without major comorbidity (9). Our study, using a similar pre-defined classification of the patients showed a similar incidence of CTEPH in consecutive acute PE patients. However, eight recently published studies were included in this updated meta-analysis (24, 26–32), which predominantly included patients with acute PE survivors. By incorporating these data, we found that the incidence of CTEPH in acute PE survivors were higher than that in acute PE survivors without major comorbidity, which was not consistent with the finding of similar incidence in the two group patients in the previous meta-analysis (9). Because comorbidities including cardiopulmonary diseases, cancers or rheumatologic disorders have been associated with increased risk of CTEPH in acute PE patients, it is not surprising that acute PE patients with these comorbidities have higher incidence of CTEPH than patients without the comorbidities (1). Another meta-analysis with 15 cohorts published by Zhang et al. (10) showed an overall incidence of CTEPH of 3.13%, which is similar to our findings. However, the authors did not perform subgroup analyses according to patient characteristics, which made the interpretation of the results difficult. Although the authors noticed a higher incidence of CTEPH in Chinese patients with acute PE than Europeans (4.46 vs. 2.82%), the different between the subgroup was not statistically significant. And ethnic differences may also affect the epidemiology of CTEPH. Compared with American patients, the high incidence of Japanese female patients (2:1 ratio) and the high incidence of chronic thromboembolism patients indicate differences in some etiological factors (12). Compared to this meta-analysis, our updated meta-analysis included four additional European studies (14, 18, 21, 28) and three additional Asian studies (30–32), and showed that a significant higher incidence of CTEPH after acute PE in Asians than Europeans (5.08 vs. 1.96%). Of note, the Asian studies were from developing countries such as China, Iran, and Thailand. The relative low healthcare resources and poor patient compliance may increase the proportion of high-risk PE patients that visited the clinical follow-up (40), which may partly explain the higher incidence of CTEPH in patients of acute PE in these Asian countries compared those from Europe.

Different from the previous meta-analysis, a comprehensive subgroup analyses were performed in our meta-analysis, which demonstrated that factors regarding study design may also significantly affect the reported incidence of CTEPH in patients after acute PE. We found that incidence of CTEPH is higher in retrospective cohorts than prospective cohorts, and in small-scale studies than large-scale studies. Retrospective studies may be confounded by recall bias, and small-scale cohort studies, as compared with multicenter prospective cohort studies, could be affected by inherited limitations of selection bias. Therefore, including retrospective and small-scale cohort may overestimate the incidence of CTEPH after acute PE as shown by our results. Besides, we found that screening of CTEPH in all PE patients or selected PE patients did not seem to significantly affect the reported incidence. These findings suggest that close monitor of the symptoms related to CTEPH is important for early diagnosis of CTEPH in patients after acute PE, and screening CTEPH for symptomatic patients after PE may be adequate. However, this is challenged by patients with chronic thromboembolic disease (CTED), who may present with normal pulmonary hemodynamics at rest despite symptomatic disease (41). Moreover, using TTE for primary screening appeared to be associated with a reduced incidence of CTEPH as compared with studies using V/Q lung scan, although the result did not reach statistical significance. In view of the potential advantages of V/Q lung scan as the methodology of choice to exclude CTEPH compared to TEE (42), as well as the inadequate use of V/Q lung scan for CTEPH in current clinical practice, a screening modality incorporating V/Q lung scan for CTEPH should be performed.

Our stratified analyses showed that previous VTE and unprovoked PE were both significantly associated with increased risk of CTEPH after acute PE. Although a previous meta-analysis also suggested these two risk factors, the authors did not perform quantitative analyses (10). Our results confirmed these findings by showing that patients with previous VTE or unprovoked PE had 2.57 and 2.71 folded risk of CTEPH than those without previous VTE or with provoked PE. Acute PE patients with previous VTE are considered as high-risk and recurrent PE is significantly associated with CTEPH (43). As for patients with unprovoked PE, the underlying disease is not identified and treated (44), which may also expose these patients to recurrent PE and CTEPH. Close monitor of symptoms related to CTEPH in these high-risk patients should be performed for the early diagnosis of CTEPH.

Our study has limitations which should be considered when interpreting the results. Firstly, both retrospective and prospective cohort studies were included, and the sample sizes of the included studies were generally small. Multicenter, prospective cohort studies with good quality remain needed to determine the exact incidence of CTEPH after acute PE. Secondly, studies included for some subgroup analyses were limited. Moreover, due to the univariate characteristics of the analysis, results of subgroup analyses should be interpreted with caution. Thirdly, the anticoagulation therapy after diagnosis of acute PE may affect the incidence of CTEPH. Since data regarding the anticoagulation status of the included patients were rarely reported, we were unable to determine its influence on CTEPH incidence. Finally, meta-analyses evaluating the association between previous VTE, unprovoked PE and the risk of CTEPH in patients after acute PE were based on univariate data from observational studies. These associations may be affected by confounding factors, and multivariate based analyses should be performed to confirm the results. However, due to the relatively low incidence of CTEPH, these studies are expected to have large sample size.

## Conclusion

In conclusion, this updated meta-analysis demonstrated that CTEPH after acute PE is not rare and with an incidence of ~3% and a higher incidence exists in Asians than in Europeans. Patient characteristics and study design factors may affect the incidence of CTEPH. Efforts should be made for the early diagnosis and treatment of CTEPH in PE patients, particularly for high-risk population.

## Data Availability Statement

The original contributions presented in the study are included in the article/Supplementary Material, further inquiries can be directed to the corresponding authors.

## Author Contributions

ZZhai conceived, designed, or planned the study. WP, ZZhan, ZW, KZ, MZ, YZ, and SZ collected and assembled the data. WP, ZZhan, QG, SZ, XT, JW, and WX performed or supervised analyses. WP and ZZhan wrote sections of the initial draft. All authors provided substantive suggestions for revision, reviewed and approved final version of the paper, and for all aspects of the work in ensuring that questions related to the accuracy.

## Funding

This study was funded by Chinese Academy of Medical Sciences (CAMS) Innovation Fund for Medical Sciences (CIFMS) (No. 2018-I2M-1-003); National Natural Science Foundation of China (No. 81970058). This study received funding from MSD China Holding Co. Ltd. The funder had the following involvement with the study: editorial assistance.

## Conflict of Interest

The authors declare that the research was conducted in the absence of any commercial or financial relationships that could be construed as a potential conflict of interest.

## Publisher's Note

All claims expressed in this article are solely those of the authors and do not necessarily represent those of their affiliated organizations, or those of the publisher, the editors and the reviewers. Any product that may be evaluated in this article, or claim that may be made by its manufacturer, is not guaranteed or endorsed by the publisher.

## Abbreviations

CTEPH: chronic thromboembolic pulmonary hypertension
PE: pulmonary embolism
VTE: venous thromboembolic events
CTPA: computed tomographic pulmonary angiography
V/Q: ventilation-perfusion
PH: pulmonary hypertension
NOS: Newcastle-Ottawa Scale
SEs: stand errors
OR: odds ratio
CI: confidence interval.
