# Supplementary material for: Higher Incidence of Chronic Thromboembolic Pulmonary Hypertension After Acute Pulmonary Embolism in Asians Than in Europeans: A Meta-Analysis
Source: Front Med (Lausanne). 2021 Oct 26;8:721294. doi: 10.3389/fmed.2021.721294 (PMC8575791; doi:10.3389/fmed.2021.721294)
Supplement: Supplementary file 1 [file Table_1.DOCX]

**Supplementary Table 1** Comparisons between studies used in the three meta-analyses evaluating the incidence of CTEPH after acute pulmonary embolism after acute PE

| **Original cohort studies** | Ende-Verhaar et al. 2017 | Zhang et al. 2018 | Pang et al. 2021 |
| --- | --- | --- | --- |
| Pengo V, Lensing AW, Prins MH, Marchiori A, Davidson BL, Tiozzo F, et al. Incidence of chronic thromboembolic pulmonary hypertension after pulmonary embolism. *N Engl J Med*. (2004) 350:2257-64. | **√** | **√** | **√** |
| Becattini C, Agnelli G, Pesavento R, Silingardi M, Poggio R, Taliani MR, et al. Incidence of chronic thromboembolic pulmonary hypertension after a first episode of pulmonary embolism. *Chest*. (2006) 130:172-5 | **√** | **√** | **√** |
| Miniati M, Monti S, Bottai M, Scoscia E, Bauleo C, Tonelli L, et al. Survival and restoration of pulmonary perfusion in a long-term follow-up of patients after acute pulmonary embolism. *Medicine (Baltimore)*. (2006) 85:253-62 | **√** | **√** | **√** |
| Klok FA, van Kralingen KW, van Dijk AP, Heyning FH, Vliegen HW, Huisman MV. Prospective cardiopulmonary screening program to detect chronic thromboembolic pulmonary hypertension in patients after acute pulmonary embolism. *Haematologica*. (2010) 95:970-5 | **√** |  | **√** |
| Martí D, Gómez V, Escobar C, Wagner C, Zamarro C, Sánchez D, et al. Incidence of symptomatic and asymptomatic chronic thromboembolic pulmonary hypertension | **√** | **√** | **√** |
| Poli D, Grifoni E, Antonucci E, Arcangeli C, Prisco D, Abbate R, et al. Incidence of recurrent venous thromboembolism and of chronic thromboembolic pulmonary hypertension in patients after a first episode of pulmonary embolism. *J Thromb Thrombolysis*. (2010) 30:294-9 | **√** | **√** | **√** |
| Surie S, Gibson NS, Gerdes VE, Bouma BJ, van Eck-Smit BL, Buller HR, et al. Active search for chronic thromboembolic pulmonary hypertension does not appear indicated after acute pulmonary embolism. *Thromb Res*. (2010) 125:e202-5 | **√** | **√** | **√** |
| Berghaus TM, Barac M, von Scheidt W, Schwaiblmair M. Echocardiographic evaluation for pulmonary hypertension after recurrent pulmonary embolism. *Thromb Res*. (2011) 128:e144-7 | **√** |  | **√** |
| Giuliani L, Piccinino C, D'Armini MA, Manganiello S, Ferrarotti L, Balbo PE, et al. Prevalence of undiagnosed chronic thromboembolic pulmonary hypertension after pulmonary embolism. *Blood Coagul Fibrinolysis*. (2014) 25:649-53 | **√** | **√** | **√** |
| Guérin L, Couturaud F, Parent F, Revel MP, Gillaizeau F, Planquette B, et al. Prevalence of chronic thromboembolic pulmonary hypertension after acute pulmonary embolism. Prevalence of CTEPH after pulmonary embolism. *Thromb Haemost*. (2014) 112:598-605 | **√** | **√** | **√** |
| Held M, Hesse A, Gött F, Holl R, Hübner G, Kolb P, et al. A symptom-related monitoring program following pulmonary embolism for the early detection of CTEPH: a prospective observational registry study. *BMC Pulm Med*. (2014) 14:141 | **√** |  | **√** |
| Kayaalp I, Varol Y, Çimen P, Demirci Üçsular F, Katgı N, Unlü M, et al. The incidence of chronic thromboembolic pulmonary hypertension secondary to acute pulmonary thromboembolism. *Tuberk Toraks*. (2014) 62:199-206 | **√** | **√** | **√** |
| De Foneska D, Condliffe R, Elliot CA, et al. Incidence and severity of chronic thromboembolic pulmonary hypertension following the introduction of a one-stop clinic for acute pulmonary embolism. Thorax 2014; 69: Suppl. 2, A63–A64 | **√** |  | Not included as this is a conference abstract. |
| Hogele J, Gall H, Voswinckel R, et al. Long-term screening for pulmonary hypertension after pulmonary embolism. Eur Respir J 2014; 44: Suppl. 58, P2321 | **√** |  | Not included as this is a conference abstract. |
| Klok FA, Tesche C, Rappold L, Dellas C, Hasenfuß G, Huisman MV, et al. External validation of a simple non-invasive algorithm to rule out chronic thromboembolic pulmonary hypertension after acute pulmonary embolism. *Thromb Res*. (2015) 135:796-801 | **√** | **√** | **√** |
| Vavera Z, Vojacek J, Pudil R, Maly J, Elias P. Chronic thromboembolic pulmonary hypertension after the first episode of pulmonary embolism? How often? *Biomed Pap Med Fac Univ Palacky Olomouc Czech Repub*. (2016) 160:125-9 | **√** | **√** | **√** |
| Yang S, Yang Y, Zhai Z, Kuang T, Gong J, Zhang S, et al. Incidence and risk factors of chronic thromboembolic pulmonary hypertension in patients after acute pulmonary embolism. *J Thorac Dis*. (2015) 7:1927-38 |  | **√** | **√** |
| Xi Q, Wang Y, Liu Z, Zhao Z, Luo Q, Gu Q, et al. Effect of Red Cell Distribution Width on Long-term Follow-up Study in Patients With Acute Pulmonary Thromboembolism. *Chin Circ*. (2016) 31:65-8 |  | **√** | **√** |
| Xu W, Cui J, Ma H, Fu B, Yan X, Qi J, et al. Investigation of prognostic effect of red cell distribution width on chronic thromboembolic pulmonary hypertension in patients with acute pulmonary embolism. *Chin J Geriatr*. (2016) 35:581-6 |  | **√** | **√** |
| Pesavento R, Filippi L, Palla A, Visonà A, Bova C, Marzolo M, et al. Impact of residual pulmonary obstruction on the long-term outcome of patients with pulmonary embolism. *Eur Respir J*. (2017) 49. doi: 10.1183/13993003.01980-2016 |  |  | **√** |
| Coquoz N, Weilenmann D, Stolz D, Popov V, Azzola A, Fellrath JM, et al. Multicentre observational screening survey for the detection of CTEPH following pulmonary embolism. Eur Respir J. (2018) 51. doi: 10.1183/13993003.02505-2017 |  | **√** | **√** |
| Puengpapat S, Pirompanich P. Incidence of chronic thromboembolic pulmonary hypertension in Thammasat University Hospital. *Lung India*. (2018) 35:373-8. |  |  | **√** |
| Hsu CH, Lin CC, Li WT, Chang HY, Chang WT. Right ventricular dysfunction is associated with the development of chronic thromboembolic pulmonary hypertension but not with mortality post-acute pulmonary embolism. *Medicine (Baltimore)*. (2019) 98:e17953 |  |  | **√** |
| Rashidi F, Parvizi R, Bilejani E, Mahmoodian B, Rahimi F, Koohi A. Evaluation of the Incidence of Chronic Thromboembolic Pulmonary Hypertension 1 Year After First Episode of Acute Pulmonary Embolism: A Cohort Study. *Lung*. (2020) 198:59-64 |  |  | **√** |
